# Oral Health Screening for Risk Reduction for Early Periprosthetic Joint Infections of Hip and Knee Endoprostheses—Results of a Prospective Cohort Study

**DOI:** 10.3390/jcm12134451

**Published:** 2023-07-03

**Authors:** Fabian Fenske, Leah Krause, Stephan Meyer, Benjamin Kujat, Jacqueline Repmann, Michael Neuhaus, Rüdiger Zimmerer, Andreas Roth, Bernd Lethaus, Dirk Ziebolz, Gerhard Schmalz

**Affiliations:** 1 Department of Oral and Maxillofacial Surgery, University of Leipzig, 04103 Leipzig, Germany; 2 Department of Cariology, Endodontology and Periodontology, University of Leipzig, 04103 Leipzig, Germany; 3 Specialised Clinic for Orthopedics, Mediclin Waldkrankenhaus Bad Düben, 04848 Bad Düben, Germany; 4 Department of Orthopedics, Trauma and Plastic Surgery, University Hospital Leipzig, 04103 Leipzig, Germany

**Keywords:** joint replacement, periprosthetic joint infection, oral health, oral focus, interdisciplinary medicine

## Abstract

This prospective observational study had two aims: (I) to assess whether a preoperative dental screening before endoprosthesis (EP) implantation with need-based dental intervention would decrease the prevalence of periprosthetic joint infection (PJI) and (II) to evaluate whether instructed orthopedic surgeons would achieve similar results in oral screening as dentists. The preoperative oral health statuses of the patients, prior to EP insertion, were either evaluated by the patients’ general dentists (Ia) or, if the patient had not visited a general dentist, by an instructed orthopedic surgeon (Ib). Both the dentist and orthopedic surgeon used standardized risk estimation (low risk, moderate risk, and high risk) for an oral-health-related infectious complication after EP insertion, including a recommendation for further management of the patient. If required, a need-based dental rehabilitation was performed. In addition, retrospective data evaluation of a comparison group (II) was performed, which had not been screened orally preoperatively. A total of 777 patients (screening group (I): *n* = 402, of which 229 were screened by a dentist (Ia), 173 were screened by an orthopedic surgeon (Ib); comparison group (II): *n* = 375) were included. No general association between early infection rate and preoperative oral screening in general was found (1% PJI in screening group (I), 1.6% PJI in comparison group (II); *p* = 0.455). However, screening performance (dentist vs. orthopedic surgeon) had a significant impact on the prevalence of developed PJIs (*p* = 0.021). Thereby, 100% of observed infections in the screening group (I) occurred in the group with previous oral screening by an orthopedic surgeon (Ib). Furthermore, the C-reactive protein (CRP) value at discharge was significantly lower when general preoperative oral screening had been performed (group I vs. group II, *p* = 0.03). Only preoperative oral screening by a dentist had the potential to reduce oral-focus-associated EP infections; therefore, increased attention should be paid to the further promotion of interdisciplinary work between dentists and orthopedic surgeons. Dental screenings, using objectifiable criteria, as applied in this study, seem reasonable but require further validation in larger cohorts.

## 1. Introduction

Implantations and insertions of total hip and knee replacements (THR and TKR) are the most frequently performed surgical procedures in Germany [1]. More than 403,000 of these procedures were reported in 2020, and there was an increasing tendency for the next years due to demographic changes and the desire to remain mobile in old age [2,3,4]. In addition, joint replacement is a well-established surgical procedure with regularly reported and remarkable postoperative outcomes [5]. The main reasons for postoperative success are significant pain relief in comparison to the preoperative findings, functional improvement, and regained mobility and quality of life for patients [1,2].

Although postoperative success is achieved in most cases, complications following joint replacement surgery are a challenge to patients and surgeons [2]. The most common complications in this regard include aseptic loosening, dislocation of the total joint replacement (TJR), and the development of periprosthetic joint infection (PJI) [6]. A PJI is defined as “infection of tissues surrounding an artificial joint implanted in the body” [2] and occurs at a cumulative incidence of 0.7% to 1.4%, regarding hip and knee prosthesis and depending on study period, study design, and country [7,8]. Depending on the period between implantation and the onset of infection of the endoprosthesis (EP), PJI is divided into early and late infection. Early infection occurs within a period of four weeks after surgery and accounts for two thirds of the total number of infections [9]. Bacteria of the skin flora, the urogenital tract, the gastrointestinal tract, and the oropharynx are considered to be the causes of PJI [10]. Several studies address oral health as a potentially important factor influencing the development of PJI [11]. The German Society for Arthroplasty also describes the dental status as a potential risk factor for PJI [12]. The potential proportion of infections caused by oral flora pathogens range from 4% to 8% [13]. Other studies report that up to 15% of EP infections are caused by dental procedures [14].

According to a systematic review by Barrere et al. [11], the relationship between preoperative dental examination with intervention and postoperative occurrence of PJI has not yet been investigated. In previous studies dealing with preoperative screenings prior to orthopedic surgeries, preoperative dental examination has generally not been documented and, when performed, has mainly been related to periodontitis and dental abscesses. In most cases, it is neither indicated whether a dental examination was performed prior to prosthesis implantation, nor is it specified what type of dental intervention was performed. The current lack of guidelines and recommendations regarding preoperative dental screening is contributing to this problem [11], as there rarely are recommendations for preoperative dental care [15]. Barrere et al. [11] pointed out the demand for research regarding the relationship between orthopedic infections and oral health before or after orthopedic surgery, as well as the influence of time interval between the onset of an oral problem and an orthopedic infection. Moreover, Chan et al. [16] attested a great need for further research in this context. To meet these needs, the first aim of this study was to investigate whether a preoperative dental screening with need-based dental intervention would decrease the prevalence of PJI using hip and knee arthroplasty. The second aim was to investigate whether instructed orthopedic surgeons achieved similar results in oral screenings to dentists. Furthermore, recommendations for preoperative dental screenings and interventions were developed on the basis of these findings. The hypothesis for the current study was that preoperative oral screening, performed by a dentist or a trained orthopedic surgeon, with dental treatment as required, can lower the prevalence of PJI compared to a comparable cohort without dental intervention.

## 2. Materials and Methods

### 2.1. Study Set up and Design

The present study was a prospective observational study, which was performed in cooperation with the Specialized Clinic for Orthopedics Bad Düben, the Outpatients’ Department for Cariology, Endodontology and Periodontology of the University Hospital Leipzig and the Department of Oral and Maxillofacial Surgery of the University Hospital Leipzig.

The study was conducted according to the guidelines outlined in the Declaration of Helsinki. The whole study protocol was approved by the ethics committee of the University of Leipzig (No: 116/20-ek). All study participants were fully informed about the study-related anonymized data collection and analysis and gave their written consent within the general patient contract also.

### 2.2. Patients

Patients who applied for elective EP insertion (THR, TKR, and sledge) at the Specialized Orthopedic Clinic in Bad Düben were selected as participants in the screening group (I) of the study if they fulfilled the following inclusion criteria:

- planned, elective first implantation of a hip, knee, or sled prosthesis;
- surgical intervention within the study period between September 2020–March 2021;
- the presence of oral health screening (Ib).
- Study participants who fulfilled the following criteria were excluded from the study:
- Transfer of the patient to another hospital due to postoperative complications without evidence of a PJI;
- Revision surgery because of a chronic periprosthetic infection or mechanical complications;
- Wound revisions within four weeks postoperatively, without germ detection;
- Acute fracture treatment with EP;
- Exchange operations due to loosening of a previously implanted EP.

In addition, a retrospective data evaluation of a comparison group (II) was performed in a reference period (September 2019–March 2020). Thereby, 380 subjects were included in the evaluation, distributed over 208 women and 172 men (median age 70.21).

A permanent team of experienced surgeons (specialists and senior registrars exclusively) performed the planned EP insertions under the same surgical and hygienic standards during the entire period (September 2019–March 2021).

No sample size calculation was made because no prior study has investigated this issue before; due to the expected low prevalence of PJI, it was aimed to include as many patients as possible within the study period. 

### 2.3. Risk Assessment

**Figure 1:**
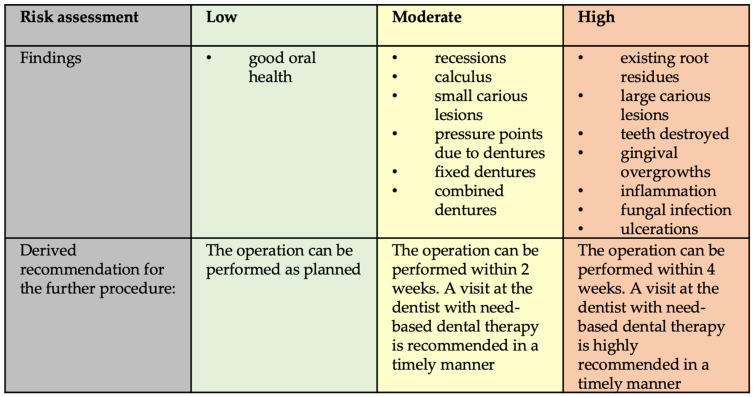
Risk assessment.

The basis of the oral screening in the current study was a risk classification system, which was applied to dentists (Ia) and orthopedic surgeons (Ib). For this, a risk stratification system was used, which has been reported previously [17]. Basically, this stratification system used equal criteria for dentists and orthopedic surgeons in the current study. In brief, the risk classification was divided into low, moderate, or high risk of developing an oral-health-related (oral focus) PJI. Thereby, oral pathologies, which mandatorily require a treatment prior to EP surgery (e.g., periodontitis apicalis, caries penetrans, remaining roots, severe periodontitis with suppuration) were classified as high risk; oral diseases that did not require urgent treatment but should be addressed after surgery (e.g., moderate periodontitis, caries media) were classified as moderate risk. Figure 1 shows an overview of the main points, which were the bases for risk assessments in this current study. 

### 2.4. Process of Data Collection

**Figure 2:**
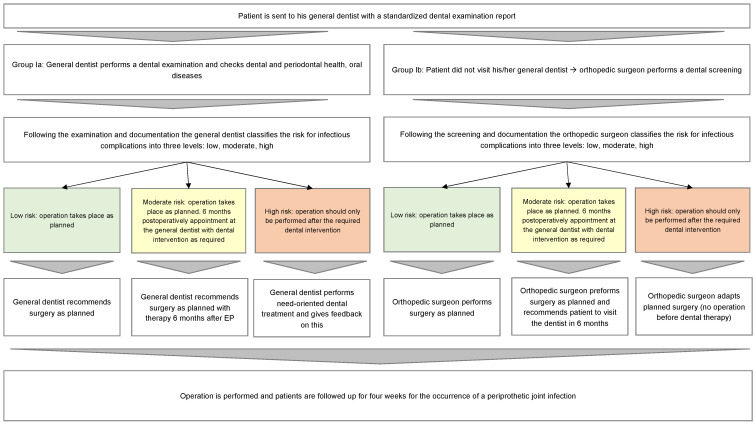
Process of data collection.

The data collection was carried out in two stages, as illustrated in Figure 2. In the first stage, the subjects’ preoperative oral health statuses were recorded. Two assessment forms were designed for this purpose: (Ia) a dental examination report, which was completed by the patient’s general dentist, and (Ib) a screening for assessing oral health. The screening (Ib) was filled out by the patient, as well as the examining doctor of the specialized clinic for orthopedics in Bad Düben. Using the two forms (Ia) and (Ib), a risk assessment of the potential oral-focus-related postoperative infection rate of the arthroplasty was made in the second stage. The risk assessment focused on the early infection, covering the first four weeks after arthroplasty insertion. A prerequisite for inclusion in the study was the screening regarding oral health (Ib). In addition, the following data were recorded using the patients’ self-reports at the start of the trial: age, gender, body mass index (BMI), nicotine consumption, diabetes mellitus status.

In the comparison group (II, *n* = 380), no additional preoperative dental measures were undertaken.

### 2.5. Dental Examination Report (Ia)

The dental examination report (Ia) was completed by the patient’s general dentist, who was instructed to perform a full oral examination. The report was used to assess oral health and to derive a standardized risk estimation for an infectious complication after EP insertion from a dental point of view. The report included the following examination items: (1) dental examination (survey for caries and filling status, detection of carious lesions requiring treatment), (2) periodontal health status (assessment of periodontal screening index, presence of perio-endo lesions), (3) presence of various oral diseases (e.g., pericoronitis, inflammatory findings in the jaw bone, diseases of the temporomandibular joint). Based on the dental findings, the risk was classified into three categories (low, moderate, high) by the general dentist, in accordance with the stratification system, which has been reported previously [17]. Moreover, need-based therapy was performed by the general dentist. Every dental intervention was fully documented by the general dentist and communicated in written form to a medical colleague from Bad Düben. The report only included the final risk stratifications of the patients from their general dentists. Therefore, the respective dentist was responsible for the risk classification. It was neither checked nor recorded whether the dentist considered patients at high risk first, treated the respective pathologies, and then included the patients into another risk group. 

### 2.6. Screening for Assessing Oral Health (Ib)

The screening for assessing oral health (Ib) consisted of two sections. The first section was completed by the patient. This section was used to collect patient-related data regarding their own oral hygiene (bleeding gums, interdental cleaning, bad taste), their dental status (tooth loosening, current complaints), and their own dental behaviors (self-assessment on gum treatments, dentist visits in the last twelve months) via a questionnaire. General medical parameters (age > 55 years, HbA1c > 6.5%) as well as the consumption of nicotine (nicotine consumption, nicotine quantity) were also collected and considered.

The second section was completed by the admitting doctor at the Bad Düben Specialized Orthopedic Clinic. This section assessed the oral health that existed preoperatively via visual examination of the oral cavity. As the admitting doctors at the clinic in Bad Düben were orthopedic surgeons, they were trained to perform a specific oral examination by a dentist before the start of the study.

Based on these examinations and questions, an ordinally scaled risk assessment was completed in the form of a traffic light system, and a recommendation for the further procedure was derived, including the criteria listed in Figure 1.

### 2.7. Statistical Analysis 

In the next step, the data were analyzed descriptively as well as interference statistically using SPSS software (SPSS Inc., Chicago, IL, USA). Metric variables were presented as statistical means and medians, whereas dispersion measures were presented as standard deviations and quartiles. Metric variables were tested for normal distribution using the Kolmogorov–Smirnov test. Categorized or nominal data were expressed as absolute and relative frequencies. For non-normally distributed samples, the Mann–Whitney U test was used as a nonparametric procedure. The categorized or nominal data, on the other hand, were analyzed using the chi-square test or Fisher’s exact test. 

Two-sided significance testing was performed for all tests, and a *p*-value < 0.05 was assumed to be statistically significant for all statistical tests.

## 3. Results

### 3.1. Patients

A total of 777 patients were included in the study. The screening group (I) contained 402 participants, divided into 219 women and 183 men (mean age 68.31 years). In addition, the retrospective data evaluation of a comparison group (II) was carried out, which included 375 patients, divided into 205 women and 170 men (mean age 70.20 years). Parameters such as gender, BMI, type of inserted prosthesis, status of the surgical wound, and comorbidities (diabetes mellitus) were comparable between both groups (*p* > 0.05, Table 1). 

The C-reactive protein (CRP) value at inpatient discharge was significantly lower in the screening group (I) than in the comparison group (II; *p* = 0.03). In addition, there were significantly more smokers in the screening group (I; *p* = 0.045), and the mean age of this group was significantly lower (*p* = 0.002; Table 1). 

### 3.2. Oral Screening of the Screening Group (I)

In the screening group (I; *n* = 402), all subjects underwent a preoperative oral examination. 56.97% (*n* = 229) of the participants were screened by a general dentist using form Ia; the remaining patients (43.03%, *n* = 173) were screened orally by a previously trained orthopedic surgeon using form Ib.

### 3.3. Dental Report (Ia)

In total, 78.6% (*n* = 180) of the 229 patients screened by a dentist and assigned to an ordinal scaled early infection risk (low, medium, high) using the examination form were assigned to low risk. A total of 21.4% (*n* = 49) of the patients were assigned to medium risk, and 0% of patients were assessed as high risk. Consequently, 23.6% (*n* = 54) of the patients underwent dental interventions, the types and extents of these are shown in Table 2.

### 3.4. Orthopedic Report (Ib)

An ordinal scaled risk assessment regarding postoperative early infection (low, medium, high) was also carried out in the group of patients examined orally by an orthopedic surgeon (*n* = 173). Here, 63% (*n* = 109) of the patients were assigned to low risk by the orthopedic surgeon, 36.4% (*n* = 63) to medium risk, and one patient (0.6%; *n* = 1) was assigned to high risk. A preoperative dental intervention was not initiated in any of the 173 study participants (Table 2).

### 3.5. PJI

In the group of the orally screened patients (I; *n* = 402), four subjects developed a postoperative early infection. This corresponded to an infection rate of 1.0%. The study participants who underwent a preoperative dental examination (Ia; *n* = 229) and the study participants who received an oral examination by an orthopedic surgeon (Ib; *n* = 173) were assessed separately (Table 3). 

In the group of patients who were examined by a dental colleague (Ia) prior to EP insertion, not a single case of postoperative early infection was recorded. The early infection rate of the 230 subjects thus amounted to 0%.

In the group of patients who were orally examined preoperatively by an orthopedic surgeon (Ib), an early postoperative infection occurred in four cases. The early infection rate of this group (Ib; *n* = 173), therefore, amounted to 2.3%. The previously assessed risk conducted by the orthopedic surgeon was "low" in three of the four patients (Table 3). 

The early infection rate was thus significantly lower following the dental examination report (Ia) compared to the screening assessment of oral health, performed exclusively by an orthopedic surgeon (Ib; *p* = 0.021, Table 3). 

In the comparison group (II, *n* = 375), six subjects developed an early infection. The resulting early infection rate of 1.6%, consequently, was higher than in the screening group (I; *p* = 0.534) but was significantly higher than in the cohort screened exclusively by dentists (Ia; *p* = 0.034; Table 3 and Table 4).

## 4. Discussion

### 4.1. Summary of Main Results

There was no general significant association between the early infection rate and preoperative oral screening. However, there was a clear accumulation of early infections that was dependent on who performed the screening. With previous oral screening by an orthopedic surgeon (Ib), 100% of the observed infection cases occurred, whereas screening by a dentist led to 0% of the cases (Ia).

In addition, the CRP value at discharge was significantly lower when preoperative oral screening was performed (I).

### 4.2. Comparison with Published Data

This is the first study investigating the effect of preoperative oral screening by both dentists and orthopedic surgeons, including documentation on the prevalence of PJI in THR, TKR, and sledges. The study included the documentation of the preoperative screening, an ordinally scaled risk assessment by an instructed orthopedic surgeon or a dentist, as well as the documentation of dental-need-based intervention.

Although the role of oral disease in the development of early infections is discussed controversially, there are several hypotheses for such an association. As we investigated the relationship between oral disease and early infection in the present study, we assumed the mechanism of an acute exacerbation of chronic oral inflammation during the early healing phase after total joint replacement [18,19,20].

Due to the restricted available data, comparability with previous studies is limited. Despite the ongoing scientific discussion concerning the oral origin of bacterial colonization of arthroplasty in the literature [21,22], there is a lack of adequate studies investigating and documenting the impact of preoperative oral screening on the prevalence of PJI. It is commonly accepted that the mouth poses a potential risk area for exogenous bacterial colonization [22]. This has led to the aforementioned hypothesis that the preoperative examination, and thus local reduction in bacterial load might, consequently, lower the risk of infection rate. However, the evaluation of the study did not support such a causal relationship: the screening group (I), being the group with preoperative examination, did not show a significantly lower early infection rate than the comparison group (II) without preoperative examination. Nevertheless, an association of infection rate with respect to the performer of the preoperative screening was observed. When oral screening was performed by a supervised orthopedic surgeon, 100% of the recorded early infections occurred in this screening group (Ib). When oral screening was performed by a dentist, 0% of the recorded early infections occurred (Ia). Thus, all cases of early infection in the screening group (I) were preceded by oral screening by an orthopedic surgeon (Ib).

This result calls for critical consideration and can be explained by a possible gap in process as well as in competence:

*Competence gap*: Given the different training, dental expertise and the motivation for a dental assessment are unequally distributed between orthopedic surgeons and dentists. The dental expertise required for the qualified oral screening is usually imparted through a university education separate from the study of human medicine, which an orthopedic surgeon has not additionally undergone as part of their training. Additionally, there is no literature dealing with the different competences regarding oral examination between orthopedic surgeons and dentists.

*Process gap*: Given the difference in equipment, the process of oral diagnostics is unequal between dentists and orthopedic surgeons. While orthopedic surgeons focused their oral diagnostics on a purely visual examination of the oral cavity, dentists were able to examine the periodontium much more intensively and, consequently, use more indicators to assess the risk of an early infection. For example, inflammation at the apical regions of teeth or periodontitis are considered oral focal points for bacterial loads. To assess such focal points, steps such as imaging diagnostics (dental X-ray, orthopantomogram, digital volume tomography), specific dental diagnostics with periodontal probes to measure bleeding on probing, and probing depths are needed, which are usually not available to an orthopedic surgeon. In addition, orthopedic surgeons are at a disadvantage in risk assessment because even simple special instruments such as dental instruments, magnifying glasses, and CO_2_ snow are not part of a classic orthopedic set-up.

Consequently, outsourcing dental assessments to orthopedic surgeons does not appear to be suitable for deriving prognostically relevant risk assessments for PJI from the results of the assessments.

In terms of risk assessment, less than 1% of high-risk classification seems to be a very low evaluation in general in the entire screening group (I), both in the orthopedically (Ib) and dentally (Ia) screened and assessed group. In comparison to cohorts of other studies, causes should thus be scrutinized critically. Schmalz et al. [17] found a rate of high-risk cases of 34% in a similar study design for potential sources of prosthetic infection based on a dental examination. This fundamental difference may have been due to a lack of communication between the surgeons and dentists who performed the screening. In contrast, surgeons and dentists in the cited study were located in one and the same university hospital, which, consequently, allowed for much closer communication and redesign of the surgical schedule. Thus, dentists, who performed the screening in the present study may have shied away from being held responsible for the non-adherence to a surgical schedule and the associated financial losses. As a consequence, dentists may have adjusted their risk assessments to the post-interventional dental statuses.

Regardless of who performed the preoperative oral screening, the CRP value after discharge was significantly lower in the screening group (I) than in the comparison group (II).

Looking at the CRP value within the screening group (I), there is only a tendency for the group screened by the dentist (Ia) to have a lower CRP value than the group screened by the orthopedic surgeons (Ib). Regarding the CRP value, it can therefore be said that the preoperative screening was successful despite the competence and process gaps on the parts of the orthopedic surgeons. However, since even chronic inflammatory diseases of oral origins such as severe periodontitis have only a questionable influence on the CRP values in blood [23], it would be conceivable that such extended surgical interventions may influence the immune system and, consequently, chronic inflammatory processes can no longer be compensated for.

It is important to reproduce this result in a larger cohort and to differentiate the cause. In any case, the lower CRP value in the orthopedically examined group (Ib) is inferior to the clinical success of the reduction in early infection rates through the screenings carried out by dentists (Ia).

In addition to the striking difference in those performing the screening, the results contradict the recommendation discussed in the literature to perform preoperative oral screening only in high-risk patients [24]. Factors such as smoking, obesity, an elevated BMI, diabetes mellitus, and male gender are considered to increase the primary risk of PJI [2]. It is known that diabetes mellitus has a potential effect on surgical results, as it affects metabolism and immune status [2]. However, the current study did not show a relevant role of diabetes, although the statistical results are strongly limited by the low number of PJI in the current study. Similarly, the influence of gender and smoking status cannot be fully assessed in the current study; thus, it remains unclear whether the difference in the current study (between Ia and Ib) would have influenced the current findings. It was found that not only the mean BMI for the patients suffering from PJI (31.20 ± 5.65) but also the mean BMI for the entire study cohort was significantly elevated (29.83 ± 5.59), as a BMI of >25 is defined as overweight, and a BMI of >30 manifests as obesity [25]. Consequently, the study cohort could generally be considered as a risk group. However, 100% of the occurring early infections were identified in non-smokers, and 80% of these in men. On closer inspection, this high BMI for the study cohort is not surprising. One of the goals of EP insertion is to regain mobility [26]. Persistent pain in previous years and, consequently, reduced mobility and movement lead to an increased BMI compared to patients with pain-free joints. In addition, the joints of patients who already have a high BMI are exposed to higher mechanical stress and are, therefore, predisposed to EP insertion [27]. Following Frey’s [24] recommendations not to perform a general dental examination prior to joint replacement but only to perform preoperative oral screening for high-risk patients would, consequently, lead to a dental examination of 729 of the 777 patients in this study, i.e., 93.82% of the study cohort, based on BMI and gender. The authors do not agree with the argument of cost-effectiveness and amount of time required that Kohler et al. [28] used to advocate for selective screening depending on existing individual risk factors and characteristics. In the designed, standardized protocol of assessment and documentation, there was no additional effort compared to the half-annual recommended dental check-ups.

*Clinical relevance and implications for practice:* PJI leads to an enormous burden, regarding reduced quality of life, pain, immobility, mortality, and financial efforts [2,6]. Accordingly, the avoidance of as many infections as possible is strongly needed, whereby oral-health-related risk factors play a relevant role, as they can be a potential source of infection [11]. Several working groups underline the need for oral examination prior to EP surgery [11,15,16,17], whereby an appropriate strategy is missing. This current study showed that preoperative screening is possible by both orthopedic surgeons as well as dentists, whereby only screening by a dentist appears to have the potential to avoid infection; therefore, the following clinical implications can be achieved through the present study:

1. Even if no significant minimizations of the early infection rates in the screening group (I) could be achieved through oral screening, a significant reduction in the occurrence of PJIs could be observed when this was carried out by dentists (Ia), making a dental screening using a risk classification and examination protocol such as in the current study recommendable.
2. Interdisciplinary cooperation must be clearly structured and documented more objectively. Detailed dental instruction of orthopedic surgeons only leads to positive tendencies, but assessments from other disciplines must at least be critically scrutinized (Ib). Therefore, the dentist should play a substantial role in the preoperative care of patients prior to EP to prevent oral-health-related PJI.

The review by Young et al. [29] also followed these demands and underlined the need for standardized protocols and recommendations for a dental check-up before THR/TKR insertion. Sendi et al. [13] also called for a dental visit prior to EP implantation with need-based focal restorations, but they criticized the lack of concepts for implementation also.

The authors, therefore, recommend a replication of the present study with a significantly greater sample size and the performance of oral screening purely by dentists with the corresponding competences and process reliabilities for the requirements of oral screenings.

### 4.3. Strengths and Limitations

This is the first study of a cohort with a retrospective comparison group that investigated the rate of PJI between a group of patients (I) receiving fully documented preoperative dental screenings prior the implantation of an arthroplasty (THR, TKR, sledge), subsequent risk classifications, and need-based interventions and a comparison group (II) without preoperative dental screenings, classifications, and interventions.

The sample size seemed comparably large, with 402 participants in the screening group (I) and 375 participants in the comparison group (II). However, due to the occurrence of PJI in only 1.3% of cases, the number of cases was very small, and, therefore, it was difficult to detect and evaluate meaningful significance. As no sample size had been performed previously, the statistical power of the sample remains questionable. The compared groups did not differ regarding comorbidities, preoperative laboratory parameters, and the orthopedic intervention itself (THR, TKR, sledge). The only significant preoperative differences between the compared groups were age and smoking behaviors. However, as 100% of the patients with early infections were non-smokers, this difference could be considered irrelevant to the study’s outcome. The higher mean age of the comparison group (II) could be considered irrelevant also, as the mean age in the screening group (I) was higher when early infections occurred. However, other heterogeneities between the groups need to be mentioned. Above all, the different recording period of the study groups remains a limiting factor. This is especially relevant against the background of the COVID-19 pandemic, which fell into the study period. It would have been reasonable to record both groups during the same period, even if the conditions in the special clinic, including the surgical team, remained unchanged throughout the study period. Nevertheless, the non-significant result between the two study groups themselves could be seen as confirmation of these conditions remaining the same. In addition to the significantly lower CRP value at discharge, the mean time interval between the intervention and the operation (29.04 ± 24.56 days) could be regarded as further confirmations of good implementations and interdisciplinary cooperations in the senses of the study’s protocol, which indicate that the medical recommendation of the study protocol (at least 14 days) was adhered to, despite the fact that operations may have already been scheduled, and thus economic interests on the part of the specialized hospital occurred. Moreover, further methodological issues need to be addressed: the exclusion criteria of the wound revision up to 4 weeks postoperatively with no evidence of germs could be seen as a possible bias. With a sensitivity of 60–80% and a specificity of 97%, the detection of germs in joint punctate should not be underestimated in the diagnosis of PJI [30].

Furthermore, due to the design of the dental examination report, how to assess the risk of an infectious complication with an oral cause remains at the discretion of the respective dentist. This risk assessment could be objectified and standardized by assigning points for possible findings that are already listed. In the current study, it can only be speculated why a low risk was chosen in eight cases, despite the fact that extractions were performed.

## 5. Conclusions

Patients experienced PJI after arthroplasty insertion in 1.6% of the cases when no preoperative oral screening took place (II) and in 1.0% of cases when it was performed preoperatively by an instructed orthopedic surgeon or by a dentist (I). The interventions in terms of oral screenings and need-based interventions thus did not lead to significant changes in the infection rates but did to significant reductions in the CRP values at the times of discharge.

In addition, the screening carried out preoperatively only by the dentists (Ia) significantly reduced the infection rates and should, therefore, be further implemented and investigated.

In the future, emphasis should be placed on the development of objectifiable criteria in a written form for preoperative oral screenings by dentists in order to further promote interdisciplinary work between dentists and orthopedic surgeons.

## Figures and Tables

**Table 1:** Characteristics of all patients before EP insertion (accepted level of significance at *p* < 0.05, significant numbers presented in bold).

		Screening Group I (*n* = 402)	Comparison Group II (*n* = 375)	*p*-Value
Age in years (mv ± sd)		68.31 ± 9.09	70.20 ± 10.04	**0.002**
Gender (male in %, (*n*))		45.5% (183)	45.3% (170)	0.958
Prosthesis % (*n*)	THR	49.5% (199)	46.1% (173)	0.418
	TKR	42.3% (170)	43.2% (162)	
	sledge	8.2% (33)	10.7% (40)	
Status of wound % (*n*)	unremarkable	98.0% (394)	97.6% (366)	0.808
	Pico	2.0% (8)	2.4% (9)	
Diabetes mellitus II (yes in % (*n*))		15.2% (61)	16.8% (63)	0.536
Smoking habits (smoker in % (*n*))		13.7% (55)	9.1% (34)	**0.045**
BMI (kg/m^2^; mv ± sd)		29.89 ± 5.88	29.76 ± 5.28	0.984
CRP at inpatient discharge (mg/L; mv ± sd)		83.93 ± 45.99	91.41 ± 49.58	**0.030**

EP: endoprosthesis, mv: mean value, sd: standard deviation, CRP: C-reactive protein.

**Table 2:** Risk assessment and performed dental interventions following the dental (Ia) or orthopedic (Ib) report of the screening group (I) (accepted level of significance at *p* < 0.05, significant numbers presented in bold).

		Screening by Dentist (Ia) (*n* = 229)	Screening by Orthopedic Surgeon (Ib) (*n* = 173)	*p*-Value
Risk assessment % (*n*)	low	78.6% (180)	63.0% (109)	**0.001**
	moderate	21.4% (49)	36.4% (63)	
	high	0% (0)	0.6% (1)	
Dental intervention (yes in % (*n*))		23.6% (54)	0	**0.001**
	extraction	27.8% (15)		
	extraction +	3.7% (2)		
	extraction + filling	5.6% (3)		
	professional tooth cleaning	31.5 % (17)		
	professional tooth cleaning + filling	5.6% (3)		
Type of intervention % (*n*)	professional tooth cleaning + tooth pocket treatment	1.9% (1)		
	endodontic treatment	3.7% (2)		
	filling	7.4% (4)		
	tooth pocket treatment	7.4% (4)		
	tooth pocket and root canal treatment	1.9% (1)		
	root canal treatment	3.7% (2)		
Days until operation after carried out intervention (mv ± sd)		29.04 ± 24.56		

mv: mean value, sd: standard deviation.

**Table 3:** Association between developed PJI, preoperative oral screening, and risk stratification (accepted level of significance at *p* < 0.05, significant numbers presented in bold).

		Screening Group I (*n* = 403)		Comparison Group II (*n* = 380)	*p*-Value
PJI (yes in %, (*n*))		1% (4)		1.6% (6)	0.455
		Screened by dentist (Ia)	Screened by othopaedic (Ib)		
		0% (0)	100% (4)		**0.021**
Risk assessment with PJI yes (in %, (*n*))	low		75% (3)		
	moderate		25% (1)		

**Table 4:** Association between developed PJI, study group, and medical data.

		PJI in Screening Group I (*n* = 4)	PJI in Comparison Group II (*n* = 6)	*p*-Value
Age in years (mv ± sd)		73.00 ± 8.79	68.83 ± 6.71	0.522
Gender (male in %, (*n*))		50% (2)	100% (6)	0.053
Prosthesis % (*n*)	THR	75% (3)	66.7% (4)	0.778
	TKR	25% (1)	33.3% (2)	
	Sledge	0% (0)	0% (0)	
Status of wound % (*n*)	unremarkable	100% (4)	83.3% (5)	0.389
	Pico	0% (0)	16.7% (1)	
	Staph. Epidermis	75% (3)	16.7% (1)	0.076
	Staph. aureus	0% (0)	66.7% (4)	
Germ detection	Staph Epidermis + Staph Capitis	0% (0)	16.7% (1)	
	HWI, actinobacter pittii (subcutan)	25% (1)	0% (0)	
Diabetes mellitus II (yes in %, (*n*))		25% (1)	33.3% (2)	0.778
Smoking habits (smoker in %, (*n*))		0% (0)	0% (0)	
BMI (kg/m^2^; mv ± sd)		27.50 ± 3.42	33.67 ± 5.68	0.084
CRP at inpatient discharge (mg/L; mv ± sd)		107.25 ± 61.18	170.33 ± 94.22	0.286

mv: mean value, sd: standard deviation.

## Data Availability

The data that support the findings of this study are not publicly available, as they contain information that could compromise the privacy of research participants, but are available from the corresponding author F.F.
